# Interaction between alcohol dehydrogenase II gene, alcohol consumption, and risk for breast cancer

**DOI:** 10.1038/sj.bjc.6600500

**Published:** 2002-08-27

**Authors:** T Stürmer, S Wang-Gohrke, V Arndt, H Boeing, X Kong, R Kreienberg, H Brenner

**Affiliations:** Department of Epidemiology, German Centre for Research on Ageing, Bergheimer Str. 20, 69115 Heidelberg, Germany; Department of Epidemiology, University of Ulm, Helmholtzstr. 22, 89081 Ulm, Germany; Department of Obstetrics and Gynecology, University of Ulm, Frauensteige 14, 89075 Ulm, Germany; German Institute for Nutritional Research, Arthur-Scheunert-Allee 114-116, 14558 Bergholz-Rehbrücke, Germany

**Keywords:** alcohol dehydrogenase II gene (ADH2), alcohol consumption, gene-environment interaction, breast cancer, epidemiology

## Abstract

*Mae*III Restriction Fragment Length Polymorphism in exon 3 of the alcohol dehydrogenase II was assessed in serum from 467 randomly selected German women and 278 women with invasive breast cancer to evaluate the interaction between a polymorphism of the alcohol dehydrogenase II gene, alcohol consumption and risk for breast cancer. In both groups, usual consumption of different alcoholic beverages was asked for using semiquantitative food frequency questionnaires. We used multivariable logistic regression to separately estimate the association between alcohol consumption and alcohol dehydrogenase II polymorphism in the population sample and women with breast cancer. The alcohol dehydrogenase II polymorphism was detected in 14 women from the population sample (3.0%) and in 27 women with invasive breast cancer (9.7%). Frequency of alcohol consumption was independent of the genotype in the population sample. In women with breast cancer, there was a significant inverse association between the alcohol dehydrogenase II polymorphism and frequency of alcohol consumption (adjusted case-only odds ratio over increasing frequency of alcohol consumption=0.5; *P* for interaction=0.02). We observed a gene-environment interaction between the alcohol dehydrogenase II polymorphism, alcohol consumption, and risk for breast cancer. Breast cancer risk associated with alcohol consumption may vary according to the alcohol dehydrogenase II polymorphism, probably due to differences in alcohol metabolism.

*British Journal of Cancer* (2002) **87**, 519–523. doi:10.1038/sj.bjc.6600500 www.bjcancer.com

© 2002 Cancer Research UK

## 

Alcohol might very well be one of the few modifiable risk factors for breast cancer (Longnecker, 1994; Smith-Warner *et al*, 1998; Singletary and Gapstur, 2001; Willett, 2001). Despite growing interest in genetic polymorphisms of the enzymes involved in alcohol metabolism for several diseases associated with alcohol comsumption (Hines *et al*, 2001; Mccarver, 2001; Yamauchi *et al*, 2001a,b), data regarding the role of these polymorphisms as to the risk for breast cancer are sparse (Freudenheim *et al*, 1999; Hines *et al*, 2000). These polymorphisms are good candidates for gene-environment interactions (Millikan *et al*, 1995), however, because they are likely to influence the most plausible biologic mechanism linking alcohol consumption to risk for breast cancer, i.e. endogenous oestrogens.

In humans, alcohol is almost entirely metabolised by oxidative detoxification, mainly dependent on two enzymes, class I alcohol dehydrogenase (ADH) and acetaldehyde dehydrogenase (ALDH). Both enzymes exhibit genetic polymorphisms influencing the rate of conversion from alcohol to acetaldehyde and to acetate (Smith, 1986). The class I ADH isoenzymes are formed by dimeric associations of α, β, and γ subunits controlled by three separate gene loci ADH1, ADH2, and ADH3, respectively (Agarwal and Goedde, 1990). Despite recent reports regarding the ADH3 polymorphism and risk for breast cancer (Freudenheim *et al*, 1999; Hines *et al*, 2000), nothing is known about the role of the less frequent but metabolically more relevant ADH2 polymorphism and its interaction with alcohol consumption for the risk of breast cancer.

The aim of the study was to test the *a priori* hypothesis of an interaction between the ADH2-2 polymorphism alone, alcohol consumption, and the risk for breast cancer in Caucasian women.

## MATERIALS AND METHODS

We used the case-only design (Khoury and Flanders, 1996) to evaluate the interaction between the ADH2-2 polymorphism and alcohol consumption with respect to the risk for breast cancer. The case-only design has more power to assess gene-risk factor interactions than the case-control design, but its applicability depends on the independence of the genetic factor and the risk factor in the population. Therefore, the independence between the ADH2 polymorphism and frequency of alcohol consumption was first assessed in a random sample of the German population.

### Random population sample

The population sample consisted of women selected from a national health and nutrition survey conducted among healthy people in the western part of Germany in 1987/1988. The survey methodology has been previously described in detail (Schneider *et al*, 1992; Speitling *et al*, 1992). In brief, a stratified probability sample was drawn from the noninstitutionalised adult population (⩾18 years of age) of German nationality. With an overall participation rate of about 70%, 1132 women were recruited in the study. Personal interviews were conducted at the participants' homes and included detailed information on medical history, dietary habits including alcohol consumption, and various life-style characteristics. Blood samples were obtained by venipuncture after fasting overnight from 965 women (85%). Sera were stored in the gas phase of liquid nitrogen at −130°C.

### Women with breast cancer

Women with breast cancer were recruited in the context of a state-wide study on risk factors and diagnostic procedures for various forms of cancer, including breast cancer, conducted in Saarland, a state with about one million inhabitants in Western Germany. The study was approved by local and regional institutional review boards. Patients 80 years of age or younger, with a first diagnosis of invasive breast cancer between 1 October 1996 and 28 February 1998 were eligible and recruited for the study by their treating physicians during the first hospitalisation due to the cancer, typically several days to weeks after initial treatment (Arndt *et al*, 2002). Out of 458 patients deemed suitable for participation by their treating physicians, 387 (85%) fulfilled all of the inclusion criteria stated above and were willing to participate. After written informed consent, interviews following a standardised questionnaire and blood drawings were performed by trained medical doctors. The interview included detailed information on known risk factors for breast cancer, including alcohol consumption during the year preceding the onset of symptoms. Clinical and pathological characteristics of the breast cancers, including menopausal status, were abstracted from hospital records, including pathology reports. Blood samples were obtained from 288 women with breast cancer (74.4%, the remaining women did not consent to blood drawing or blood drawing was not possible) and were immediately transported to the study centre and centrifuged on the same day. Sera were stored at −80°C. For the present analysis, eight women with non-German nationality and two in whom genotyping failed were excluded.

### Genotyping

Serum samples from the population sample and women with breast cancer were analysed separately and without further information regarding the women (including their alcohol consumption) according to the same protocol in a central laboratory at the Department of Obstetrics and Gynecology of the University of Ulm. The analysis of *Mae*III RFLP was based on the PCR amplification of a fragment using the following primers: sense primer, 5′-AAT CTT TTC TGA ATC TGA ACA G-3′, and anti-sense primer 5′-GAA GGG GGG TCA CCA GGT TG-3′. All PCRs were carried out in 25 μl aliquots containing 5 μl of denaturated serum, 5 pmol of each primer, 1X reaction buffer, 50 μM dNTPs, and 0.25 U Taq polymerase. The amplification was for 30 cycles each consisting of 1 min denaturing at 94°C, 1 min of annealing at 60°C and 1 min of extension at 72°C. An initial denaturation step of 3 min at 94°C and a final extension at 72°C for 5 min were used. PCR products were digested to completion with *Mae*III at 55°C for 12 h. Digests were separated on a 12% polyacrylamide gel. All ethidium bromide-stained fragments were analysed on a UV source using an image analysis system. The ADH2-2 allele of the polymorphism was defined as the presence of the *Mae*III digestive site, according to a previous publication (Goedde *et al*, 1992).

### Alcohol consumption

In both the population sample and women with breast cancer, consumption of different alcoholic beverages was assessed using a semiquantitative food–frequency questionnaire. Women from the population sample were asked about the usual number of days per week on which they consumed different beverages as well as the usual quantity of these beverages consumed per week (Müller *et al*, 2001). Women with breast cancer were asked about the usual consumption of defined quantities of different alcoholic beverages (beer, wine, spirits) per day, week, or month in the year preceeding the onset of symptoms. In all women, the usual frequency of consumption of alcoholic beverages was categorised into less than once a week, once a week, and more than once a week according to the alcoholic beverage most often consumed.

### Statistical analysis

Out of the random population sample, we randomly selected up to two women within the same 5-year age-category for every woman with breast cancer. We then described sociodemographic factors and known or suspected risk factors for breast cancer in this age-matched population sample and women with breast cancer. The odds ratios (OR) and their 95% confidence intervals (CI) for the association between categories of alcohol consumption and the ADH2 polymorphism were estimated separately in the population sample and women with breast cancer using multivariable logistic regression. All analyses were controlled for age (continuous), body mass index (weight in kg^2^ height in m, continuous), smoking (never, ever), age at menarche (continuous), parity (0, 1, 2, 3 or more), and history of breast cancer in the mother. The case-only analyses were additionally controlled for menopausal status, age at menopause (continuous), and cumulative years of hormone replacement therapy (continuous) (Colditz and Rosner, 2000).

## RESULTS

The mean age of the population sample of 467 women was 54.9 years, slightly lower than the mean age of the 278 cases with invasive breast cancer (57.2 years, see Table 1

**Table 1:**
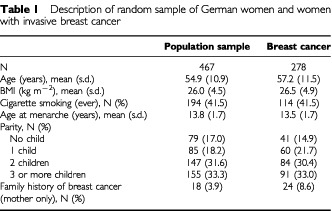
Description of random sample of German women and women with invasive breast cancer

). The majority of women with breast cancer (72.7%) were postmenopausal at the time of diagnosis. Whereas the body mass index was slightly lower in the population sample than in women with breast cancer, the prevalence of cigarette smoking (current and former) was virtually identical (41.5%). In the population sample, the mean age at menarche was 13.8 years compared to 13.5 years in women with breast cancer. About one-third of all women had given birth to two, and another third to three or more children. A family history of breast cancer in the mother was reported by 18 women from the population sample (3.9%) and 24 women with breast cancer (8.6%).

The majority of women with breast cancer were postmenopausal (72.7%) and had T1 (43.8%) or T2 (45.2%) tumours. Most cancers were ductal (74.1%) and oestrogen (70.1%) as well as progesteron (65.1%) receptor positive, and 42.1% had already metastasised to lymph nodes at the time of diagnosis (data not shown).

In the population sample, 14 heterozygotes of ADH2 (β1β2) were detected (3.0%) compared to 27 heterozygotes in women with breast cancer (9.7%, see Table 2

**Table 2:**
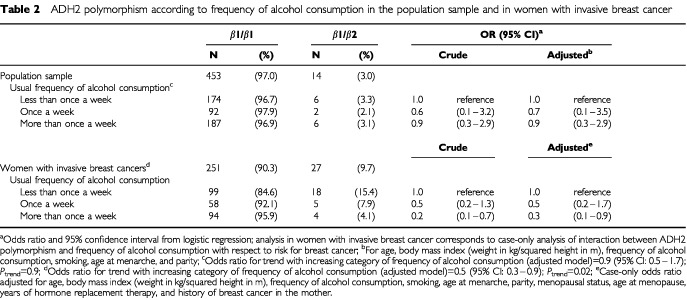
ADH2 polymorphism according to frequency of alcohol consumption in the population sample and in women with invasive breast cancer

). None of the women was β2β2 homozygote.

The proportion of women consuming alcoholic beverages less often than once a week or more often than once a week was approximately equal (around 40% each) in women from the population sample (see Table 2). The prevalence of the ADH2 polymorphism by category of alcohol consumption ranged from 2.1–3.3% and was independent of the frequency of alcohol consumption (adjusted OR for trend with increasing frequency of alcohol consumption=0.9, *P*=0.9).

Since the frequency of alcohol consumption was independent of the ADH2 polymorphism in the population sample, the possible interaction between the ADH2 polymorphism and frequency of alcohol consumption as to the risk for breast cancer could be assessed using the case-only design. During the last year preceeding the onset of symptoms, 117 women with breast cancer consumed alcoholic beverages less often than once a week and 98 women reported consumption of alcoholic beverages more often than once a week. The prevalence of the ADH2 polymorphism monotonically decreased with increasing frequency of alcohol consumption from 15.4% in women consuming alcoholic beverages less often than once a week to 4.1% in women consuming alcoholic beverages more often than once a week. The case-only analysis confirmed the inverse interaction between the ADH2 polymorphism and frequency of alcohol consumption with respect to risk for breast cancer. The adjusted case-only OR for once a week consumption of alcoholic beverages compared to women consuming alcoholic beverages less often was 0.5 (95% CI: 0.2–1.7). In women consuming alcoholic beverages more often than once a week, this OR was even more pronounced (OR=0.3; 95% CI: 0.1–0.9). There was a statistically significant trend for a lower prevalence of the ADH2 polymorphism with increasing frequency of alcohol consumption (*P*=0.02).

Additional case-only analyses revealed that the observed interaction was independent of menopausal status and any of the known risk factors for breast cancer presented in Table 1, essentially unchanged using usual amount (g day^−1^) of alcohol consumed instead of frequency of consumption, and even more pronounced (OR over increasing frequency of alcohol consumption=0.4; 95% CI: 0.2–0.9) in women with oestrogen receptor positive tumours (data not shown).

## DISCUSSION

In a population based case-only study of women with breast cancer we observed a previously unknown interaction between frequency of alcohol consumption, the ADH2 polymorphism, and risk for breast cancer.

The use of the case-only study design is dependent on the assumption that the frequency of alcohol consumption does not vary according to the genotype in the population. Since we, like others (Gilder *et al*, 1993; Whitfield *et al*, 1998; Borras *et al*, 2000), observed no association between ADH2 polymorphism and alcohol consumption in women without breast cancer, we could assess the interaction between these factors and risk for breast cancer using the case-only analysis, which is more powerful to detect gene-environment interactions than a case–control study (Piegorsch *et al*, 1994). In contrast to a case–control analysis, our analytic strategy has also the advantage of assessing associations independently in women with identical data collection procedures (i.e. first within women from the population sample and then within women with breast cancer) and therefore avoiding problems of differential misclassification often encountered in case-control studies. Furthermore, the case-only design allowed to control for menopausal status, age at menopause, and cumulative years of hormone replacement therapy in the multivariable analysis as recently suggested (Colditz and Rosner, 2000).

ADH2 is known as a key enzyme in the first step of detoxification of alcohol to acetaldehyde. The ADH2-2 allele, which encodes the more active β2-subunit, is more prevalent in Asian populations (60–80%) than Caucasians (0–10%) (Goedde *et al*, 1992) and leads to an increased metabolic rate *in vitro* (9.2 min^−1^ for β1β1 and 400 min^−1^ for β2β2 isoenzymes) (Bosron and Li, 1986). Furthermore, the ADH2 polymorphism has been shown to increase the alcohol elimination rate after alcohol consumption (Thomasson *et al*, 1993) and to influence the metabolism of alcohol in peripheral tissues (Takeshita *et al*, 1996).

Blood alcohol levels have been associated with oestradiol levels, which play a major role in the development of breast cancer (The Endogenous Hormones and Breast Cancer Collaborative Group, 2002). Although the association between alcohol consumption or blood alcohol levels and oestradiol levels in pre- (Reichman *et al*, 1993) and postmenopausal (Ginsburg *et al*, 1996, Ginsburg, 1999) women is not completely understood, the increased alcohol elimination rate in individuals with the polymorphism may lead to lower oestradiol levels and might therefore be an explanation for the observed inverse interaction. Faster clearance of alcohol might furthermore mitigate the depression of androgens and sex hormone binding globulin associated with alcohol intake (Madigan *et al*, 1998; Hines *et al*, 2000), the latter again leading to a decreased bioavailability of oestrogens.

The difference in prevalence of the ADH2 polymorphism in the population sample and in women with breast cancer is intruiging. Is the ADH2 polymorphism a previously unknown risk factor for breast cancer? This difference needs to be interpreted with caution. Women from the population sample were recruited nationwide and approximately 10 years before the recruitment of women with breast cancer. The population sample has been found to be representative of the German population (Schneider *et al*, 1992), however. We furthermore found no regional differences in the prevalence of the ADH2 polymorphism in the population sample (*P*=0.4, data not shown) and changes in the prevalence of the polymorphism over such a short time period are unrealistic. Population stratification or confounding by different genotypes, which are culturally linked to alcohol consumption, is unlikely to introduce relevant bias in this exclusively German population with very similar genetic background (Wacholder *et al*, 2000). Finally, although the polymorphism was analysed separately in both groups, the same methods were used in the same laboratory by the same technicians.

ADH has been found in different organs not directly involved in alcohol metabolism, including the breast (Saleem *et al*, 1984). In combination with xanthine oxidoreductase, ADH may lead to the formation of reactive oxygen species that themselves have been linked to types of DNA damage typically observed in breast cancer and therefore contribute to breast carcinogenesis (Wright *et al*, 1999). Furthermore, Class I ADH plays an important role in the provitamin A (beta-carotene) metabolism, oxidizing all-*trans*-retinol and 9-*cis*-retinol, and might play a role in hydroxysteroid metabolism (Duester, 2000). Therefore, there seems to be evidence that some forms of ADH may play important roles in human tissues other than the liver, including the breast.

Although we carefully controlled for potential confounding factors in our analysis, the potential for residual confounding by additional factors cannot entirely be ruled out. In particular, the observed risk and interaction may not be entirely due to the ADH2 polymorphism itself, but also to the linkage of ADH2 with other genes that are associated with an increased risk for breast cancer. ADH2 has been observed to be linked to ADH3 (Borras *et al*, 2000) and the ADH3*1 genotype was observed to be a risk factor for breast cancer in premenopausal women (Freudenheim *et al*, 1999). However, given the stronger metabolic effects of ADH2, the ADH3–breast cancer association might also be confounded by ADH2.

In summary, we observed a previously unknown interaction between alcohol consumption and the ADH2 polymorphism with respect to risk for breast cancer. If replicated, the observed gene-environment interaction might be a good example for the future need of a concept for tailor-made preventive recommendations taking individual genetic susceptibilities into account. It might, however, also be a good example for the complexity and difficulties in applying such recommendations in practice due to the low prevalence of the ADH2 polymorphism in the caucasian population.
